# Step-by-step surgical procedures for a correct identification of the sentinel lymph node in endometrial cancer

**Published:** 2021-01-08

**Authors:** s Restaino, A Finelli, A Lucidi, A Ercoli, G Scambia, F Fanfani

**Affiliations:** Fondazione Policlinico Universitario A. Gemelli IRCCS, UOC di Ginecologia Oncologica, Dipartimento per la Tutela della Salute della Donna e della Vita Nascente, L.go A. Gemelli; 00167 Roma, Italia; Gynecologic Oncology Department of Medicine and Aging Sciences University “G. d’Annunzio”of Chieti-Pescara, Via dei Vestini 131, 66100 Chieti (CH), Italy; Gynecologic Oncology, Università degli Studi di Messina, Piazza Pugliatti, 1, 98122 Messina ME, Italy; Università Cattolica del Sacro Cuore, Roma Largo A. Gemelli, 1 - 20123 Milano, Italia

**Keywords:** sentinel lymph node, endometrial cancer, laparoscopy

## Abstract

**Background:**

In patients with endometrial cancer, the common method for assessing the status of lymph nodes (LN) is lymphadenectomy. The sentinel lymph node (SLN) biopsy is a revolutionary concept and it will play an increasingly important role in surgical practice. The surgical technique of the sentinel lymph node is less destructive than lymphadenectomy, and it requires less anatomical knowledge.

**Methods:**

Step by step technique of cervical injection, the preparation of the anatomical spaces and the identification of the main structures to detect and remove the SLN safely in patients affected by endometrial cancer stage IA.

**Results:**

We identify the three different lymphatic pathways drainage from the uterine cervix and show how anatomical retroperitoneal knowledge is essential for the safe dissection of anatomical spaces. In literature it is reported that in about 9% of cases the SLN is located at the lumbo-aortic level, so it is clear how important it is to know the anatomy to follow the highlighted lymph pathway to identify first lymph node absorber of the drainage.

**Conclusion:**

Anatomical knowledge and the correct preparation of the anatomical spaces make the identification of the sentinel lymph node safe and feasible.

## Introduction

Endometrial cancer (EC) is the most common carcinoma of the female reproductive organs in developed countries with a lifetime risk of 2.8% (National Cancer Institute Surveillance, Epidemiology and End Results Program, 2020), with over 300,000 new cases diagnosed each year worldwide. Historically, standard treatment consists of surgery (total hysterectomy, bilateral salpingo- oophorectomy with or without pelvic and aortic lymphadenectomy) for apparent uterine-confined endometrial carcinoma (NCCN Guidelines ® , 2020; NCCN Guidelines ® , 2015). The use of lymphadenectomy for endometrial carcinoma is consistent with various observational studies which confirmed a high percentage (22%) of occult metastatic disease when removal and pathological analysis of lymph nodes from pelvic and para- aortic basins was applied (Creasman et al., 1987). Several randomised controlled trials have identified that lymphadenectomy alone does not improve survival for women with endometrial carcinoma in low-risk populations and when nodal status is not used to determine adjuvant therapy, and yields only prognostic information, rather than providing direct therapeutic benefit (Frost et al., 2017). Today, the role of lymphadenectomy and its extension is still being debated in the scientific community. However, given the known importance of systemic adjuvant therapy for node-positive patients, and the poor reliability of clinical assessment of nodes, pathologic nodal evaluation remains imperative (Randall et al., 2006). Additionally, lymphadenectomy is associated with morbidity including intra and post-operative complications, with a notable depletion in the quality of life of the patient (Hareyama et al., 2015). Currently, SLN biopsy is considered a standard in the treatment of melanoma and breast cancer. SLN was first described in 1996 by Burke et al. (1996) for endometrial cancer. The European Society of Gynaecological Oncology (ESGO) - European Society for Medical Oncology (ESMO) - European Society for Radiotherapy and Oncology (ESTRO) and the National Comprehensive Cancer Network (NCCN) approved the sentinel lymph node (SLN) mapping algorithm in 2015 (ESMO-ESGO-ESTRO Consensus Conference on Endometrial Cancer, 2020) for the staging of endometrial cancer. The concept of SLN biopsy refers to the selective and directed sampling of the first-in-chain lymph nodes that drain from a malignant tumour.

Recently, evaluation of feasibility for high-risk or intermediate-risk endometrial cancer patients has been reported in a multi-centre study. They suggest that the SLN algorithm did not decrease oncologic outcomes compared with systematic pelvic and para- aortic lymphadenectomy, but today the indication for SLN biopsy in high-risk patients is controversial (Buda et al., 2018; Schlappe et al. 2018).

## Methods

### 

**Figure 1:**
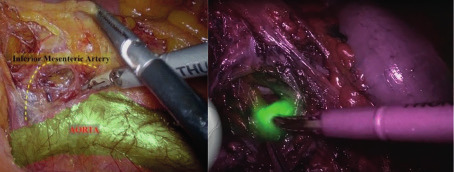
SLN at the level of the inferior mesenteric.

We reported two patients, both 55 year-olds with endometrial cancer stage I. Preoperative workup included bimanual pelvic examination, pelvic ultrasound, magnetic resonance imaging (MRI) and computer tomography (CT). The ultrasound and MRI reported a <50% myometral infiltration, with no CT signs of distant localisation. The patients were discussed by the oncological team, following a previously validated algorithm. The procedures were performed by the same team. We performed a laparoscopic total hysterectomy with bilateral salpingo-oophorectomy with SLN evaluation. The extemporaneous examination, in both the cases, of the uterus and SLN gave a stage IA. One of the four SLN was identified at the level of the inferior mesenteric artery following the supraureteral paracervical pathway (Figure 1).

The patient, under general anaesthesia, is positioned in the dorsal lithotomic position with both legs supported in stirrups, with a Trendelenburg tilt and arms along the body. Four sterile trocars are used. A 12 mm port is inserted at the umbilicus for the telescope. Once pneumoperitoneum (12 mmHg) is achieved, intra-abdominal visualisation is obtained with a 0° high- definition telescope. Three additional 5 mm ports are placed under direct visualisation, in the right lower abdomen medial to the right obliterated umbilical artery and in the left lower abdomen lateral to the inferior epigastric vessels. One more 5-mm trocar is inserted in the mid abdomen at the level of the umbilicus.

### 1. Evaluation:

As a first surgical step we proceed to evaluate the organs and peritoneum of the upper abdomen for any heteroplastic lesions or adhesions. The pelvic organs, the uterine morphology, the presence of heteroplastic lesions and the possible adhesions present are evaluated.

### 2. Cleaning:

As a surgical approach before the injection at the level of the cervix, it is essential to proceed with the so-called “preparation” of the pelvic operating field, removing all the adhesions between the internal female genital organs and the colon/ rectum. The intention of this manoeuvre is to allow greater mobility of the uterus and allow easier and direct access to the retroperitoneum.

### 3. Injection:

**Figure 2:**
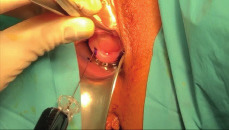
Cervical injection of Indocyanine green.

After adhesiolysis, we perform a cervical injection, of green Indocyanine (ICG) solution at the 3 and 9 o’clock position using a total of 4-5 ml (Figure 2). A 25 mg vial with ICG powder is diluted in 20 mL of aqueous sterile water. For each side, 1 ml is injected 1 cm into the stroma and 1 ml at superficial level, in order to obtain a precise mapping of the lymphatic course. We prefer to practice injection with a spinal anesthesia needle (27 gauge) (Restaino et al., 2017). The operator in charge of the injection communicates to the first operator the side (according to the patient) and time of inoculation; same procedure for the contralateral side.

### 4. Opening:

After about 20 minutes from the first inoculation, the retroperitoneum is accessed bilaterally, after coagulation and section of the round ligament and extension of the peritoneal window on the anterior and posterior leaf of the broad ligament. At this point, if the injection has been satisfactory, one (or more rarely two) of the three lymphatic pathways will be clearly coloured, which leads to a lymph node group.

### 5. Development:

Once the first dye lymph node of the chain has been identified, being careful not to confuse the dilated lymphatic vessels with lymph nodes, the pelvic spaces are developed. The aim is to identify the ureter and the origin of the uterine artery, allowing the application of clips or coagulation of the same in total safety for a better hemostatic control, as we previously described (Gueli Alletti et al., 2020).

### 

The pararectal spaces are entered between the ureter and the internal iliac artery. This manoeuvre allows for the identification of the uterine artery as it leaves its origin from the internal iliac artery.

**Figure 3:**
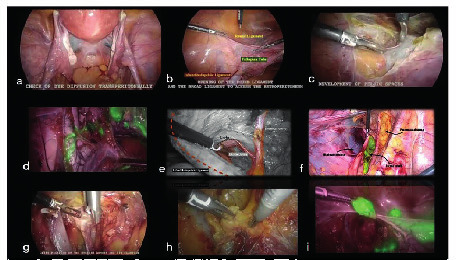
Step-by step demonstration. a: Check the dye diffusion. b: Identification of the round ligament and of infundibolopelvic ligament. c: Coagulation and cut of the round ligament. d: Identification of lymphatic route. e/g: Developing the pelvic space and identification of the uterine artery and ureter. g: Clipping of the uterine artery. h/i: Identification and withdraw of the sentinel lymph node.

- The medial pararectal space (Okabayashi’s space) is developed between the mesoureter and the rectouterine ligament by opening up a space between the posterior leaf of the broad ligament (medial) and the ureter (lateral).
- The lateral pararectal space (Latzko’s space) is developed between the mesoureter and pelvic wall by opening up the space between the internal iliac artery (lateral) and the ureter (medial).
- Paravesical Spaces: their point of separation being the medial umbilical ligaments (obliterated umbilical arteries), laterally by the obturator internus muscle and the obturator nerve, artery, and vein; the posterior border (toward the sacrum) is the endopelvic fascial sheath around the internal iliac artery and vein and its anterior branches, as they course towards the ischial spine. The pubocervical fascia forms the floor of this lateral compartment as it inserts into the arcus tendineus fasciae pelvis (fascial white line) (Figure 3).

After identification, the sentinel node is removed; as far as possible we try to never take the lymph node directly with laparoscopic instruments, but to manage it through the present circumferential fat, and send it for histological examination. If a SLN is not identified on both sides of the pelvis, a side-specific complete lymphadenectomy should be performed on the unmapped side as described and proposed by Barlin et al. (2012) , to reduce the incidence of false- negative SLN.

## Results

**Figure 4:**
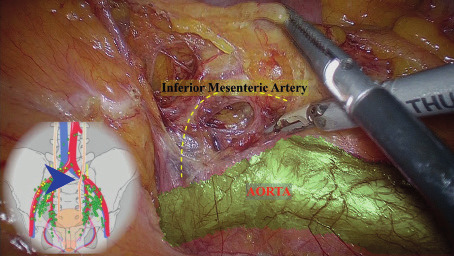
Supraureteral paracervical pathways and infraureteral paracervical pathway .

**Figure 5:**
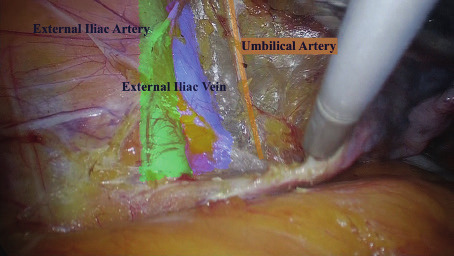
Neural paracervical pathway.

Three different lymphatic pathways have been identified as described by Ercoli et al. (2010): the supraureteral paracervical pathways, the infraureteral paracervical pathway (Figure 4) and the neural paracervical pathway (Figure 5). In particular:

- The supraureteral paracervical pathway, limited cranially by the peritoneum and caudally by the ureter (this group of lymph vessels runs into the paracervix, particularly into the connective mesenteries enveloping the uterine artery, the superficial uterine vein, and their branches which form the superficial portion of the vesicouterine ligament).
- The infraureteral paracervical pathway, located between the ureter, cranially, and the deep uterine vein, caudally (the infraureteral paracervical lymph channels ended into the interiliac and inferior gluteal/pudendal nodes)
- The neural paracervical pathway formed in both cases by 1 lymph channel emerging from the utero-vaginal fascia below the origin of the deep uterine vein, and ending into the interiliac and inferior gluteal/pudendal nodes in proximity of the ischiatic spine. It run into the caudal portion of the paracervix comprised between the deep uterine vein and the pelvic floor, which contains the inferior hypogastric nerve and plexus, and their efferent branches directed towards the pelvic organs.
- The mean operative time was 79 minutes , blood lost 100cc and both patients were discharged after 48 h. No post-operative complications were reported for up to 6 months.

The timing taken in the beginning of the dissection and detailed knowledge of retroperitoneal anatomy allows one to perform the sentinel lymph node technique even when due to anatomical variations, the first nodal lymph node, following the lymph node diffusion pathway, may be present in the extrapelvic region.

If we do not visualise the sentinel lymph node in pelvis, we are obliged to look for it at the lumbo- aortic level, precisely because we know that in about 9% of cases the SLN could be extrapelvic (Collarino et al.,2016).

## Discussion

The incidence of lymph node metastasis in patients with clinical Stage I or II endometrial cancer is approximately 10%, suggesting that lymphadenectomy may be unnecessary in most patients with early-stage endometrial cancer (Niikura et al., 2019).

Lymph node metastasis was reported in 25 of 425 (5.9%) patients with preoperative Grade 1– 2 endometrioid carcinoma with <50% invasion who underwent SLNB (Khoury-Collado et al., 2016). Zahl Eriksson et al. (2016) reported that the SLN technique allowed increased identification (16.7% vs. 7.3%) of Stage IIIC1 disease, even though a lower median number of lymph nodes were removed, compared to selective lymphadenectomy. This is due to the ultrastaging that increases the detection of micrometastases. The SLN technique alone resulted in a lower incidence of leg lymphoedema compared to infrarenal para-aortic and pelvic lymphadenectomy (Leitao et al., 2020).

Niikura et al. (2019) suggest that it is necessary to perform evaluation of para-aortic lymph node metastasis for exact endometrial cancer staging .

In accordance with the data in the literature, even in our centre we currently prefer to use ICG technique compared to other tracers because it is more accurate (Restaino et al., 2017; Buda et al., 2016; Papadia et al., 2017). Furthermore, ICG increases the probability of SNL detection compared to blue dye by approximately 26.5% (Rozenholc et al., 2019). Ballester et al. (2011) with their SENTI-ENDO study, reported 100% sensitivity and negative predictive value of the SLN per hemi- pelvis, but when analysed at the patient level the sensitivity dropped to 84% . Rossi et al. (2017) with their FIRES (Fluorescent Imaging for Robotic Endometrial cancer Sentinel lymph node biopsy) study estimated that the technique was associated with a sensitivity of 97.2%, negative predictive value of 99.6%, and a false-negative rate of 2.8% .

Participating surgeons of the FIRES study were asked if the identified SLNs were located in chains that were within or outside of the traditional boundaries of lymphadenectomy. Approximately 17% of identified SLN’s were located outside of traditional lymphadenectomy basins with a similar proportion of patients with stage IIIC disease having nodal metastases identified exclusively in these atypical regions which would have been overlooked by conventional node dissections. How et al. (2017) also evaluated the presence of “atypical” lymph node locations, and they found sentinel lymph nodes in regions of the pre-sacrum, internal iliac, and parametrial locations, outside of traditional node dissection boundaries . These lymph nodes in atypical locations represented 17% of all observations. Ten percent of patients with node-positive disease contained disease only in sentinel lymph nodes found in atypical regions, and therefore only detected with the sentinel lymph node procedure.

Surgeons are recommended to closely evaluate the para-aortic nodal basins for both SLNs or clinically suspicious nodes as part of the SLN algorithm. We firmly believe, that in the light of the scientific literature present, the sentinel lymph node technique should be a prerogative only for gynaecologists with proven knowledge of the retroperitoneum and adequate training in surgical techniques.
